# Peatland warming strongly increases fine-root growth

**DOI:** 10.1073/pnas.2003361117

**Published:** 2020-07-13

**Authors:** Avni Malhotra, Deanne J. Brice, Joanne Childs, Jake D. Graham, Erik A. Hobbie, Holly Vander Stel, Sarah C. Feron, Paul J. Hanson, Colleen M. Iversen

**Affiliations:** ^a^Environmental Sciences Division, Oak Ridge National Laboratory, Oak Ridge, TN 37830;; ^b^Climate Change Science Institute, Oak Ridge National Laboratory, Oak Ridge, TN 37830;; ^c^Department of Geosciences, Boise State University, Boise, ID 83725;; ^d^Earth Systems Research Center, University of New Hampshire, Durham, NH 03824;; ^e^Kellogg Biological Station, Michigan State University, Hickory Corners, MI 49060;; ^f^Department of Physics, Universidad de Santiago de Chile, Santiago, 9170022, Chile;; ^g^School of Earth, Energy and Environmental Sciences, Department of Earth System Science, Stanford University, Stanford, CA 94305

**Keywords:** peatland, belowground plant response, experimental warming, elevated carbon dioxide, fine roots

## Abstract

Belowground climate change responses remain a key unknown in the Earth system. Plant fine-root response is especially important to understand because fine roots respond quickly to environmental change, are responsible for nutrient and water uptake, and influence carbon cycling. However, fine-root responses to climate change are poorly constrained, especially in northern peatlands, which contain up to two-thirds of the world’s soil carbon. We present fine-root responses to warming between +2 °C and 9 °C above ambient conditions in a whole-ecosystem peatland experiment. Warming strongly increased fine-root growth by over an order of magnitude in the warmest treatment, with stronger responses in shrubs than in trees or graminoids. In the first year of treatment, the control (+0 °C) shrub fine-root growth of 0.9 km m^−2^ y^−1^ increased linearly by 1.2 km m^−2^ y^−1^ (130%) for every degree increase in soil temperature. An extended belowground growing season accounted for 20% of this dramatic increase. In the second growing season of treatment, the shrub warming response rate increased to 2.54 km m^−2^ °C^−1^. Soil moisture was negatively correlated with fine-root growth, highlighting that drying of these typically water-saturated ecosystems can fuel a surprising burst in shrub belowground productivity, one possible mechanism explaining the “shrubification” of northern peatlands in response to global change. This previously unrecognized mechanism sheds light on how peatland fine-root response to warming and drying could be strong and rapid, with consequences for the belowground growing season duration, microtopography, vegetation composition, and ultimately, carbon function of these globally relevant carbon sinks.

## Significance

Peatlands store up to two-thirds of the world’s soil carbon, but this carbon may be released under warmer conditions, creating an important climate feedback. The belowground warming response of peatlands is particularly uncertain even though factors such as plant root growth regulate ecosystem water, carbon, and nutrient cycles. We studied how peatland fine roots respond to warming in a whole-ecosystem experiment. Fine-root growth increased dramatically, +130% for a degree of warming, primarily driven by soil drying. This warming response is 20 times stronger than in other ecosystem experiments, highlighting peatland vulnerability to warming. Our study elucidates large and rapid belowground changes that will affect peatlands of a warmer world and their ability to store carbon into the future.

Northern peatlands contain some of the most carbon-rich soils globally (1, 2), and their warming response could feed back to further climate warming, given that carbon (C) losses from warming soils depend on the initial ecosystem C stocks (3, 4). Our understanding of peatland responses to environmental change is based primarily on aboveground plant dynamics (5–7). Investigations of plant belowground responses to altered environmental conditions are relatively few (8, 9). In uplands, environmental changes can alter fine-root production and mortality, thereby affecting ecosystem functions such as nutrient and water uptake, ecosystem respiration, and ultimately, soil C storage (10–13). These adaptive fine-root responses are poorly understood and thus, not well represented in peatland C cycling models (14).

Ecosystem-scale experiments help define the response of ecological processes to climate change and improve the predictive power of terrestrial biosphere models spanning spatial scales from individual ecosystem processes to the entire land surface (15). We investigated fine-root response to experimental whole-ecosystem warming and elevated [CO_2_] at the SPRUCE (Spruce and Peatland Responses Under Changing Environments) experiment, in an ombrotrophic peatland in northern Minnesota, the United States. This whole-ecosystem warming experiment in a treed peatland includes both above- and belowground warming and provides a wide range of warming treatments. In 10 large open-top enclosures (7-m tall and 12.8 m in diameter), belowground warming (to 2-m depth) and air warming were initiated in 2014 and 2015, respectively. There were five temperature treatments (+0 °C, +2.25 °C, +4.5 °C, +6.75 °C, and +9 °C above ambient) and two CO_2_ treatments (ambient and elevated). Elevated [CO_2_] was initiated in June 2016 and had a target of +500 ppm above ambient (∼900 ppm) (16). In each of the 10 experimental enclosures, we used root-ingrowth cores to measure in situ new root growth and tissue chemistry between 2014 and 2017.

Along with fine-root growth responses to soil temperature, soil moisture, and elevated [CO_2_], we assessed fine-root incorporation of ^13^C-depleted C from the elevated [CO_2_] treatment, which could indicate changes in root population turnover time, and δ^15^N shifts in roots, which could indicate changes in root N source (17–19). We captured root growth across three key northern peatland plant functional types (PFTs; ericaceous shrubs, trees, and graminoid species) and two microtopographical features (hummocks and hollows) so that our treatment response functions may be compared with other sites or used to parameterize Earth System Models (20).

## Fine Roots Increase with Warming but Due to Drying

**Fig. 1.**
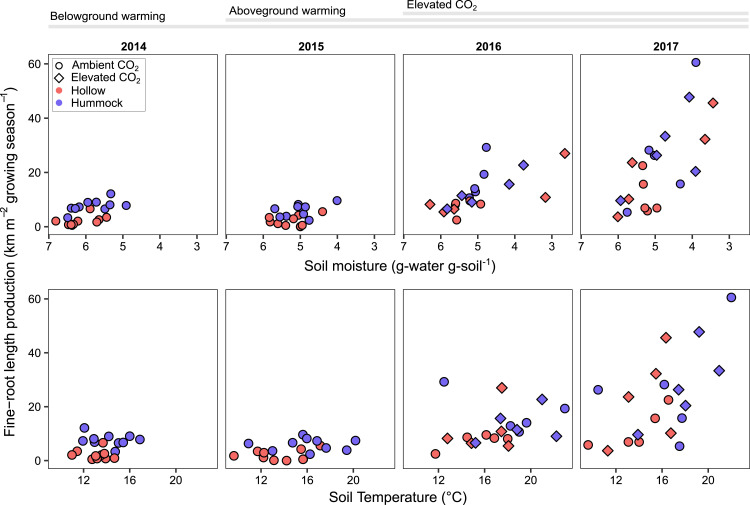
Plot-scale (PFTs summed) responses of fine-root growth to warming and drying conditions during the growing seasons of 2014, 2015, 2016, and 2017 at the SPRUCE experiment. Fine-root responses became stronger, as inferred from the ranges of the *y* axes and from the increasing slopes presented in *SI Appendix*, Table S1, as experimental treatments were added in each year (initiation of belowground warming in 2014, addition of aboveground warming in 2015, addition of elevated [CO_2_] in 2016). In a mixed effects model (Table 1), soil moisture was a significant predictor of the fine-root response. Growth was measured between June and October and represents the growing season. Soil temperatures are averages of hourly measurements at 10-cm depth from June to October. Soil moisture is from gravimetric moisture content measurements made on collected ingrowth core peat. Note that the *x* axes in the soil moisture panels range from wetter to drier conditions from left to right. Growth trends were similar for biomass production and length production (*SI Appendix*, Fig. S1). We show only length results hereafter and refer to them as fine-root growth or length production.

On the plot scale (summed across all PFTs), fine-root growth increased significantly with warming treatments but was better predicted by drying than by warming or elevated [CO_2_] (mixed effects model *R*^2^ = 0.68) (Fig. 1 and Table 1). Since soil moisture and temperature were not correlated (*SI Appendix*, Fig. S2), our analyses isolated the stronger influence of drying over warming on root growth.

**Table 1.** Mixed effects models on the plot scale or by PFT to explain variation in the fine root-length production (log transformed)

	Model-adjusted *R*^2^	Slope estimate	SE	Degrees of freedom of denominator	*P* value	Random effect variation explained, %
Plot scale (summed all PFTs)	0.68					
Intercept		4.63	1.12	25.2	0.0001	
Fixed effects						
Soil temperature		−0.01	0.03	92.5	0.3526	
Soil moisture*		−0.51	0.12	93.2	0.0001	
CO_2_ treatment		0.04	0.11	93.8	0.7610	
Random effects						
Year of sampling						37.5
Topography						28.4
Shrub	0.59					
Intercept		5.91	1.42	34.0	0.0002	
Fixed effects						
Soil temperature		−0.04	0.05	91.6	0.3557	
Soil moisture*		−0.71	0.16	91.7	0.0001	
CO_2_ treatment		−0.23	0.16	91.9	0.1462	
Random effects						
Year of sampling						27.9
Topography						22.2
Larch	0.40					
Intercept		3.15	1.93	75.3	0.1067	
Fixed effects						
Soil temperature		0.05	0.07	50.6	0.5151	
Soil moisture*		−0.87	0.26	74.5	0.0013	
CO_2_ treatment		−0.00	0.24	74.3	0.9947	
Random effects						
Year of sampling						15.5
Topography						0.811
Spruce and graminoid	NS					

We investigate the contribution of soil temperature, soil moisture, and CO_2_ treatment (ambient or elevated) as fixed effects in the model and year of sampling and topography (hummock or hollow) as random effects. A full model at the plot scale with nonsignificant and interaction terms included is in *SI Appendix*, Table S2. The fixed effect of year was also explored in the full model and did not change the dominant effect of moisture, but it highlighted that fine-root responses became stronger as the experimental treatments were initiated. NS, nonsignificant model.

* Significant effects.

Among PFTs, the fine roots of ericaceous shrubs responded most strongly to drying (mixed effects model *R*^2^ = 0.59) (Table 1). Fine roots of the deciduous conifer *Larix laricina* (larch) also increased (Table 1), while the evergreen conifer *Picea mariana* (spruce) and graminoids had no significant response.

Tree and shrub PFTs differed in their root morphology and thus, resource acquisition strategies; shrub roots had up to 20-fold higher specific root length (SRL; root length per unit root mass) than the trees (SRL mean and SE for fine roots of shrubs = 0.38 ± 0.01 km g^−1^, larch = 0.02 ± 0.001 km g^−1^, and spruce = 0.04 ± 0.002 km g^−1^ km g^−1^). Thus, thin-rooted shrubs may be more efficient in investing C per unit of root length for resource foraging compared with thicker-rooted trees (21), although shrubs and trees also differ in their association with ericoid and ectomycorrhizal fungi, respectively. While shrubs responded strongly to warming by increasing fine-root length, the more limited length response but higher biomass response of larch fine roots may be related to differing resource acquisition strategies (*SI Appendix*, Fig. S3). Shrubs may be taking a “do-it-yourself” approach by increasing the length production of new roots for foraging, while larch may be mainly “outsourcing” resource acquisition by investing in mycorrhizal fungal partners (22); both strategies have implications for total belowground C input, decomposition rates (23), and ultimately, peatland C storage. Lastly, the tree fine-root response may be mirroring aboveground C gain; parallel investigations have found that seedlings of the two tree species differ in their aboveground responses to warming (positive for larch, negative for spruce) (24). Investigations of PFT-specific root–fungal interactions and aboveground–belowground linkages in the bog are underway, but for the remainder of the manuscript, we focus on the shrub fine root-length response given that it was the strongest and earliest response and has implications for peatland C function through negative effects of shrubs on the growth of keystone *Sphagnum* mosses.

## Strong Increases in Shrub Fine-Root Growth

**Fig. 2.**
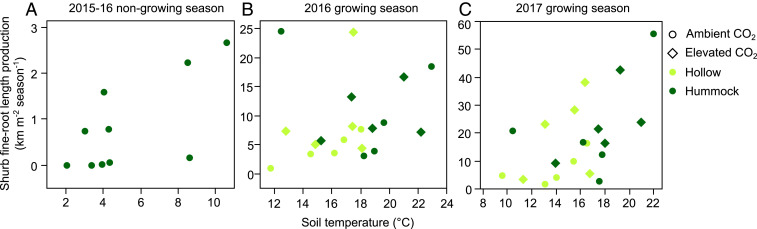
Shrub fine-root growth response (*A*) from October 2015 to June 2016 nongrowing season, (*B*) from June to October 2016 growing season, and (*C*) from June to October 2017 growing season. Note that both *x* and *y* axes have different scales in each panel. In the nongrowing season, shrub fine-root growth increased by 0.24 ± 0.09 km m^−2^ °C^−1^ (*R*^2^ = 0.45, *P* = 0.03; bivariate linear regression of root length predicted by soil temperature, *n* = 10 because only hummock cores had nonzero growth in this season and elevated [CO_2_] was not yet initiated). In the 2016 growing season, shrub fine-root growth increased by 0.96 ± 0.28 km m^−2^ °C^−1^ where *R*^2^ = 0.52 and *P* = 0.01 (full factorial regression of root length predicted by soil temperature and CO_2_ treatment). In the 2017 growing season, shrub fine-root growth increased by 2.54 ± 0.87 km m^−2^ °C^−1^ where *R*^2^ = 0.32 and *P* = 0.01 (full factorial regression of root length predicted by soil temperature and CO_2_ treatment). Data are shown for both hummocks and hollows and ambient and elevated [CO_2_] treatments in the growing season. In 2016, *n* = 18 instead of 20 because the two highest-growth outliers were removed from the regression but are shown in B; these two points are not outliers when regressed against moisture as they were the driest plots as shown in *SI Appendix*, Fig. S4*A*.

During the growing season, shrub fine-root growth increased with warming (Fig. 2*B*) or drying (*SI Appendix*, Fig. S4*A*). We also quantified fine-root growth outside of the typical growing season (October to June; henceforth termed nongrowing season); it was zero in 2014 but increased in the warmed plots after whole-ecosystem warming began in 2015 (Fig. 2*A*). This additional growth between autumn and spring suggests an extension of the belowground growing season due to warming.

Our data suggest that for every degree increase in soil temperature, shrub fine-root growth increased by 0.96 ± 0.28 km m^−2^ in the 2016 growing season (*R*^2^ = 0.52, *P* = 0.01) (Fig. 2*B*) and by 0.24 ± 0.09 km m^−2^ in the nongrowing season (*R*^2^ = 0.45, *P* = 0.03) (Fig. 2*A*). In the growing season of 2017, shrub root warming response was even stronger with a 2.54 ± 0.87-km m^−2^ °C^−1^ increase (*R*^2^ = 0.32, *P* = 0.01) (Fig. 2*C*).

Thus, relative to the shrub fine-root growth in the control plot (0.9 km m^−2^ y^−1^) (+0 °C and ambient [CO_2_] plot in Fig. 2*B*), our data suggest a linear annual increase of 1.2 km m^−2^ y^−1^ for every degree increase in soil temperature, at least within the +0 °C to 9 °C applied experimental warming range (using the conservative estimate of 2016 rather than 2017 when the slope more than doubles). The magnitude of this response (130% increase of fine root-length production for a degree increase in temperature) is much higher than upland estimates of a 58% increase in fine-root biomass averaged across ∼30 warming and elevated [CO_2_] experiments (25) or a 7.1% increase in belowground productivity per 1 °C warming estimated from over a thousand manipulative studies (26).

Rapid adaptation of shrub root-length production (and thus, resource acquisition) in response to environmental change could be one mechanism by which shrubs outcompete other peatland PFTs, especially *Sphagnum* mosses—key peatland ecosystem engineers driving the accumulation of carbon in peatlands—under warmer and drier conditions. This “shrubification” of peatlands in response to warming or drying has been reported previously (5), but mechanisms of this phenomenon are poorly understood (27). While *Sphagnum* moss growth is negatively influenced by shrubs primarily via shading (28), the influence of shrubification on other PFTs such as trees could be positive. Peat drying and increases in nutrients could increase tree recruitment and growth (29), and we see some evidence of this in the increased larch fine-root biomass (*SI Appendix*, Fig. S3) and aboveground growth (24).

**Fig. 3.**
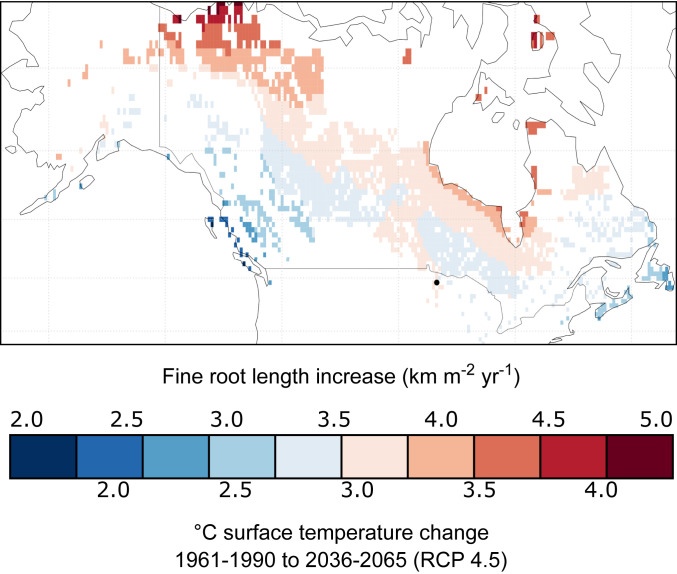
Hypothetical increases in shrub fine root-length production given expected temperature increases (based on RCP 4.5) in northern peatlands (North America example). We used the 2016 linear model of 1.2-km^−2^ m^−2^ y^−1^ increase in shrub fine-root length per degree warming to calculate the potential changes in peatland shrub root growth. The RCP 4.5 scenario was based on the mean annual surface temperature projected by the MMM of 10 GCMs. Note that not all these peatland regions contain ericaceous shrubs, but since we do not have a map of how species ranges may change in the future, we could not mask out regions without shrubs. *SI Appendix*, Table S4 has details on models used. The black dot shows the location of the SPRUCE site.

Given the potential for ericaceous shrubs to rapidly change the C balance of peatland ecosystems through cascading effects on other species, we focus next on the broad-scale implications of the shrub fine-root warming response. In the northern peatlands of North America, based on RCP 4.5 (Representative Concentration Pathway 4.5; CO_2_ stabilization scenario), an average warming of 3 °C is expected by midcentury (ranges shown in Fig. 3). Given these temperature scenarios, we extrapolated a simple estimate of potential increases in shrub fine root-length production (Fig. 3). We recognize that this approach is an oversimplification because not all peatland areas contain shrubs; for example, fens typically contain few or no ericaceous shrub species (30). Furthermore, since we do not know future shrub occurrence across peatlands, we simply show the ranges of expected future temperatures and corresponding potential shrub fine-root growth across all peatlands in North America (a similar approach could be used on northern peatlands globally). While our extrapolation assumes a linear response of shrub fine roots to warming based on our data (Fig. 2), we can envision nonlinear responses as the SPRUCE warming treatment continues. The most plausible scenario would be that at a temperature threshold, moisture would become too limiting and shrub fine-root production would decrease after an initial increase. Future work could apply our temperature response parameters to mechanistic models and improve extrapolations.

## Drivers of Shrub Fine-Root Responses

Similar to our findings at the whole-plot scale, shrub fine-root response was better predicted by drying than warming (Table 1). One possible reason for fine-root growth change is increased belowground allocation to fine roots relative to aboveground production of stems and leaves due to a moisture or nutrient limitation (31). We expected drying to increase the ratio of shrub fine-root length to aboveground biomass (31) and saw this response in 2016 (*SI Appendix*, Fig. S4*C* and Table S3). However, we expected the opposite pattern with increasing nutrient availability (31); instead, the ratio of fine-root growth to aboveground biomass increased with increasing nutrients (*SI Appendix*, Fig. S5 shows increasing nutrients with warming, inferred from ion-exchange resins), again highlighting the relative importance of drying over warming or over increases in nutrient availability (8, 31–35). Increases in root growth may also be driven by increased aerobic peat space as the water table lowers with drying. Raised hummocks were drier and had more roots than lower-elevation hollows prior to warming (Fig. 1) (11). However, root growth in hollows increased strongly after drying in these previously inundated areas (Fig. 1 and *SI Appendix*, Fig. S6). Increased shrub abundance in hollows could raise them up to hummocks and could flatten the existing microtopography, influencing peatland resilience to environmental change (36–38).

## Warming and Elevated [CO_2_] Influence Peatland Nutrient Cycling

We also assessed fine-root tissue chemistry (results from 2015 and 2016) to further elucidate mechanisms of belowground responses to climatic change. Plant isotopes can reflect differences in nutrient availability and acquisition strategies among PFTs and in response to environmental change (19, 39–41).

Warming and elevated [CO_2_] influenced peatland nitrogen cycling. Shrub fine-root tissue δ^15^N increased strongly with warming in 2015 (*R*^2^ = 0.93, *P* = 0.0075) (*SI Appendix*, Fig. S7*A*). Increased tissue δ^15^N could be due to a shift in N acquisition by fine roots to deeper layers given the strong ^15^N gradient with depth at the SPRUCE (42), particularly given the observed drying of the aerobic layer (*SI Appendix*, Fig. S6). It could also be due to increased N mineralization rates and subsequent plant uptake (18) in warmed plots (inferred from both increasing fine-root growth and from measurements of plant-available N assessed with ion-exchange resins as shown in *SI Appendix*, Fig. S5); N availability was also a significant predictor of shrub tissue δ^15^N (*SI Appendix*, Fig. S8). Increased N availability could also potentially decrease dependence on mycorrhizal acquisition, which could also increase δ^15^N (19, 39). Lastly, continued increase in the growth of ericaceous shrub roots with warming could mean an increased abundance of ericoid mycorrhizal fungi (ERM). Shifts to an ERM-dominated system could result in increased peat decomposition rates via greater production of phenol-oxidizing enzymes (43) or decreased peat decomposition rates as ERM compete with saprotrophic fungal decomposers (the “Gadgil effect”) (44).

The addition of elevated [CO_2_] beginning in 2016 may have increased plant N limitation, as reflected by lower shrub root δ^15^N and higher C:N relative to the ambient CO_2_ plots (*SI Appendix*, Fig. S7 *B* and *F*, respectively). This trend also matches the trend of higher shrub fine-root growth in the elevated [CO_2_] plots relative to ambient (Fig. 2 *B* and *C*) and is supported by evidence that labeled new photosynthate was quickly incorporated into new shrub fine roots within the same growing season (*SI Appendix*, Fig. S7*D*). As the SPRUCE treatments continue, we expect to see more responses in the elevated [CO_2_] plots and interactions with warming: for example, a deepening of fine-root distribution driven by nutrient limitation and not just moisture limitation (11, 12).

## Implications

Our study revealed strong and rapid belowground responses to whole-ecosystem peatland warming via drying, especially by ubiquitous understory shrubs. Peatland drying has led to shrub encroachment in previous mesocosm or open-top chamber warming experiments (45, 46) and our study elucidates belowground mechanisms of this change. Our results suggest the higher fine-root plasticity of thinly rooted shrubs over other PFTs (21) as a possible cause for rapid peatland shrubification in response to warming (via drying). Continued warming and increased aboveground shrub cover at the SPRUCE (47) may decrease the abundance of peat-producing *Sphagnum* species, loss of which has already been observed (48), and thus, decrease long-term C storage capacity of the peatland because *Sphagnum* litter decomposes more slowly than shrub litter (49).

Our results may reflect a short-term (first 4 y of experiment), initial response of the ecosystem. Nevertheless, we elucidate mechanisms that have longer-term implications: a rapid (within a year of full treatment) and strong (over 130% increase for a degree of warming) adaptive response of fine roots, variable response of plant functional and microtopography types, and the dominance of moisture feedbacks over temperature or nutrient feedbacks. An extension of the belowground growing season is also an important ecosystem response that is in line with observations on extended aboveground growing season (50). Lastly, our results provide magnitudes and ranges of fine-root responses that are required to model peatland structure and function in a warmer world.

## Methods

### Site Description.

The SPRUCE experiment is located in the S1 bog (47°30.4760′N; 93°27.1620′W; 418 m above mean sea level) at the Marcell Experimental Forest in northern Minnesota, the United States. This site is considered especially vulnerable to climate change because it is at the southern boundary of the boreal region. The S1 bog is an ombrotrophic peatland (primarily precipitation fed with minimal influence from groundwater) that receives 768 mm of precipitation annually and has an annual average air temperature of 3.3 °C (1961 to 2005 averages) (51).

The SPRUCE was established in a regrowth treed bog that had the tree layer removed in 1974. All studied trees have since regenerated through natural processes (5- to 8-m current tree height). The dominant tree species at the site are *P. mariana* (Mill.) B.S.P. and *L. laricina* (Du Roi) K. Koch (referred to as spruce and larch, respectively, in the text). At nearly 45 y postcutting, the tree overstory is still progressing through postharvest succession to the closed canopy forest that was present preharvest. In the understory, there is a near-continuous ground layer of *Sphagnum* mosses, dominated by *Sphagnum angustifolium *(*C.E.O. Jensen ex Russow) C.E.O. Jensen* and *Sphagnum fallax *(*H.Klinggr.*) *H.Klinggr*. in hollows and *Sphagnum magellanicum Brid.* on hummocks. *Pleurozium* and *Polytrichum* species are also present. A shrub layer overlying the moss layer is dominated by ericaceous shrubs including *Rhododendron groenlandicum* (Oeder) Kron & Judd and *Chamaedaphne calyculata* (L.) Moench. The herbaceous species *Maianthemum trifolium* (L.) Sloboda and various graminoid species including *Rhynchospora alba* (L.) Vahl, *Eriophorum vaginatum *L., and *Carex* species are also present. The site has microtopography with average height differences between raised hummocks and depressed hollows ranging between 13 and 17 cm (52). Pretreatment investigation of fine-root distribution and dynamics in the S1 bog indicated that fine roots were primarily in the top 30 cm of peat and there were more fine roots in hummocks than in hollows (9).

### Experimental Setup.

At the SPRUCE experiment, deep peat (2-m depth) and combined deep peat and air warming were initiated in 10 large-scale enclosures in June 2014 and June 2015, respectively. Each treatment plot is an octagonal enclosure with an open top to allow precipitation, measuring 7-m tall by 12.8 m in diameter with a mean area of 114.8 m^2^ (16). Experimental warming was achieved for five temperature targets (+0 °C, +2.25 °C, +4.5 °C, +6.75 °C, and +9 °C above ambient) and was homogenous within the enclosure boundaries (16). Elevated [CO_2_] treatments were initiated in June 2016 and had a target of +500 ppm above ambient (∼900 ppm). The added CO_2_ had a δ^13^C value of approximately −54‰ (relative to atmospheric CO_2_ of −8‰).

A sheet pile corral spanning down to the glacial till and protruding 0.5 m above the peat surface was installed around each enclosure with an outflow system to allow natural lateral water flow from each enclosure (53). Thus, water table fluctuations for each enclosure are similar to those of an independent ombrotrophic system (i.e., the water table is a function of incoming precipitation and evapotranspiration driven by temperatures of the applied treatment and is independent from those of the larger bog area).

### Fine-Root Growth.

The ingrowth core approach was used to capture newly grown fine roots during two time periods (from June to October and from October to June) in 2014, 2015, and 2016 and from June to October for 2017. Paired hummock–hollow ingrowth cores constructed of rigid polypropylene mesh and filled with moist, root-free commercial peat were installed in the 10 experimental enclosures. To ensure that the peat within the ingrowth cores represented similar conditions to the surrounding bog peat, we constructed ingrowth cores with similar bulk density (0.1 g cm^−3^). We also checked that carbon and nitrogen contents of the commercial peat were similar to the surrounding peat. Average values for bog peat within the top 30 cm were 1.1% for N and 48.5% for C (54), while commercial peat averages were 0.97% for N and 48.9% for C.

Ingrowth cores were snugly placed into holes made using a modified hole saw to 10-cm depth below adjacent hollow surface in the hummocks and to 30-cm depth below peat surface in the hollows. Cores were collected and replaced with similarly constructed cores in June and October of each year. Because the actual “growing season” length varies among temperature treatments (50), our measurements do not represent traditional growing and nongrowing seasons. However, for simplicity, we refer to our sampling periods as growing (June to October) and nongrowing (October to June) seasons in the text.

Upon removal from the peat, ingrowth cores were frozen at −20 °C until processing. Frozen cores were sectioned into 10-cm increments and thawed in the refrigerator (1 to 3 d), and all fine roots (<2 mm in diameter) were methodically removed using forceps with the aid of jeweler’s glasses. We separated the living fine roots of each tree species (*P. mariana* and *L. laricina*; spruce and larch, respectively) from multiple ericaceous shrub species simply identified as “shrubs” (shrub roots were indistinguishable from one another) and graminoid species. We refer to these groups as PFTs (shrub, graminoid, spruce, or larch). Roots were defined as alive or dead based on color and how brittle they were. All of the analyses in this paper are of the live “fine roots,” which include both the most distal roots, orders 1 to 3 (the “absorptive” fine roots), as well as orders 4 and above (“transport” fine roots) up to 2 mm in diameter (55). After fine roots were removed from the peat and cleaned with distilled water, we scanned them at 1,400 dpi on an Epson Perfection V700 Photo Scanner (Model J221A; Epson America Inc.) and quantified root length and average diameter using WinRhizo software (Regent Instruments Inc.). Fine roots were then oven dried for at least 3 d at 70 °C and weighed to determine biomass. Fine-root growth values were calculated using both length and biomass summed for each 10-cm-depth increment on a length or grams of root per meter squared of soil per sampling period basis by accounting for peat volume and soil bulk density. Growth was calculated for each of the four plant types (spruce, larch, shrub, and graminoid) and topography (hummock and hollow) within each treatment enclosure. Our analyses focus on fine-root growth by length production rather than biomass (although trends were similar in both) (*SI Appendix*, Fig. S1).

The installation and removal of ingrowth cores disturb the belowground system so the fine-root growth rates presented here may not reflect rates from an undisturbed volume of soil. Our growth rate estimates do, however, allow us to compare the relative impact of warming and elevated [CO_2_] treatments. The roots within ingrowth cores are also newly produced, which aids in interpretation of tissue chemistry response.

### Fine-Root Tissue Chemistry.

Dried fine roots were analyzed for total C and N, C:N, δ^13^C, and δ^15^N on an isotope-ratio mass spectrometer (Integra CN; SerCon). Glucose (δ^13^C = −10.2‰) and ammonium sulfate (δ^15^N = −0.4‰) were the working standards for the δ^13^C and δ^15^N analyses, respectively, and were tested against NIST 1515 (apple leaves) and NIST 1575a (pine needles). Isotopes are expressed in the delta notation ([*R*_sample_ − *R*_standard_]/*R*_standard_) × 1,000, where *R* is the ratio of ^13^C:^12^C or ^15^N:^14^N.

### Environmental Data.

For each plot, we calculated soil temperature averages using half-hourly measurements taken 10 cm below the hollow peat surface (“B” temperature measurements in the dataset) (56). This 10-cm depth was chosen because 90% of the roots were expected to be above this depth (9). Soil temperature data were averaged for the periods during which ingrowth cores were in the peat profile. These measured (rather than target treatment) temperature values were used in all our statistical analyses (57). To assess soil moisture at each core location, we used a relative measure that was generated directly from the ingrowth cores after their retrieval from the field. Cores were frozen and kept in sealed bags. Upon thawing, we calculated gravimetric water content (grams of water per gram of soil; hereafter referred to as soil moisture) for each core section using a 5-g subsample of the peat. While this soil moisture is integrated over the period of incubation, it will likely be biased by the moisture level nearest to the retrieval date.

### Fine-Root and Aboveground Allocation (Shrub Only).

We used shrub canopy volumes measured using terrestrial laser scanning (TLS) as a proxy for aboveground shrub biomass allocation. Previous studies have demonstrated strong correlations between TLS-derived “voxel volumes” and aboveground biomass (e.g., refs. 58–62), which make voxel volume a well-suited metric for quantifying aboveground biomass surrounding our root ingrowth sampling locations. While we estimated both shrub and tree aboveground biomass separately, we only present shrub results in this study as tree results were not statistically significant.

To estimate our proxy of aboveground shrub biomass, voxel volumes were calculated from TLS point clouds taken with a Riegl VZ-1000 3D Terrestrial Laser Scanner (Riegl Laser Measurement Systems) (63). For each plot, four TLS scans were collected from the interior boardwalk of the SPRUCE plots (16) and subsequently georegistered to produce one point cloud per plot per year from 2015 to 2016. TLS scans were taken in July and August when foliage was at or near peak. To calculate voxel volumes, the point clouds were broken into 0.01-m three-dimensional volumetric pixels (i.e., voxels). The number of voxels with at least one TLS return representing shrubs, and which were within a 0.5-m radius of the ingrowth core locations, was summed to produce the final voxel volumes. These volumes, therefore, represent the quantity of shrub biomass within 0.5 m of where the fine roots were sampled. For ease of reading and interpretation, these canopy volumes are henceforth referred to as aboveground biomass, and the ratio between fine-root growth and aboveground biomass is used as a proxy for fine root:aboveground allocation.

### Plant Available Nutrients.

Ion-exchange resin capsules (WECSA, LLC) were used to monitor in situ changes in plant-available nutrients in aerobic and anaerobic peat layers at approximately monthly intervals during the growing seasons of 2014 to 2016. Resin-access tubes were installed in hummock–hollow pairs at each plot (9). At each collection, resin capsules (UNIBEST International) were removed and replaced, and removed resins were extracted with 2 M KCl, filtered with a Whatman #1 filter, and kept frozen at −20 °C until thawed and analyzed for NH_4_^+^ on a Lachat autoanalyzer (Hach Company). For our analyses, we averaged monthly plant-available NH_4_-N between June and October. We averaged 2016 data from hummock and hollow resins and used the 10- and 30-cm depth samplings in each microtopographic location to focus on the depths targeted for root ingrowth.

### Extrapolation over North American Northern Peatlands.

For Fig. 3, we used monthly mean surface temperature data rendered by 10 Global Climate Models (GCMs) (*SI Appendix*, Table S4 shows additional details) from the Coupled Model Intercomparison Project 5 (64). Data from the GCMs were obtained from the World Climate Research Program’s Working Group for the Coupled Model Intercomparison Project–Phase 5 (see https://esgf-node.llnl.gov/projects/esgf-llnl/) and regridded to a common 0.5° × 0.5° grid. We calculated the changes, from 1961 to 1990 to 2036 to 2065, in the multimodel mean (MMM) of the surface temperature according to the RCP 4.5 scenario (i.e., the RCP that stabilizes radiative forcing at 4.5 W m^−2^ in the year 2100) (65). Although several ensembles are available for some models, the MMM of the temperature was computed by using estimates rendered by the first ensemble of each model. This approach aimed to avoid assigning more weight to one model over the other.

The increase in root length estimated in peatlands over North America is based on the change, from 1961 to 1990 to 2036 to 2065, in MMM of the surface temperature according to the RCP 4.5 scenario. The peatland area was adopted from the PEATMAP project (66) after regridding to the same 0.5° × 0.5° grid used for the GCMs. The extrapolation used the slope derived from the regression analysis between root growth and temperature in the conservative year (2016; slope = 1.2 km fine roots m^−2^ y^−1^ °C^−1^).

### Statistical Analyses.

Analyses were first conducted at the plot scale (PFTs summed) to gain an ecosystem-scale understanding and then, individually for each PFT. Growth analyses presented are on fine root-length production, but biomass showed similar trends (*SI Appendix*, Fig. S1 shows length and biomass comparisons). The two topographical features within each plot were kept as individual data points to capture the range of temperatures and moistures in the hummocks and hollows (analyzed as random effects in predictive models). After finding the strongest fine-root growth responses in shrubs, fine-root growth and comparison with aboveground biomass analyses focused on only 2016 and 2017 shrubs, given that these years had both warming and elevated [CO_2_]. The fine-root chemistry analyses were conducted on 2015 and 2016 for each of the plant types.

To evaluate the effect of the experimental treatment on fine-root growth (first plot scale, then for each plant type), log-transformed fine root-length production was predicted using mixed effects linear models. Fixed effects were soil temperature, CO_2_ treatment, soil temperature × CO_2_ treatment, and soil moisture. The interaction effect was always nonsignificant and thus, removed in the reported best-fit models. In addition to topography as a random effect, all mixed effects linear regression models included year of sampling as a random effect (given that 2014 and 2015 had incomplete experimental treatments) (*Experimental Setup* has details). A full model with nonsignificant terms as well as the fixed effect of year is also shown in *SI Appendix*, Table S2 as a supplement to Table 1.

To understand mechanisms behind fine-root growth responses, we investigated both above-/belowground responses as well as root tissue chemistry. We analyzed fine-root growth from the most responsive plant type (shrubs) in the full treatment year (2016) as a ratio to aboveground biomass to investigate whether below-/aboveground allocation is responding to soil temperature (nonsignificant) or moisture. We then investigated the moisture effect using mixed effects models for fine-root growth, aboveground biomass, and a ratio of the two (fixed effects were soil moisture and CO_2_ treatment, and the nonsignificant temperature effect was removed from the model; random effect was topography).

For fine-root tissue chemistry, we evaluated warming or drying response of fine-root tissue percentage C and N, C:N, δ^13^C, and δ^15^N using bivariate linear regression models with either moisture or temperature as a predictor and ran a model for each sampling year, PFT, topographical feature, and elevated [CO_2_] treatment. Only the significant models are reported in the results. The unreported models were nonsignificant due to either high variability or missing data (shortage of root-tissue material to analyze for chemistry). Low degrees of freedom due to the missing data also made it difficult to use mixed effects models similar to previous analyses to investigate chemistry responses while taking into account the random effects of topography and year of sampling.

### Data Accessibility.

All data used in this paper are archived at and available from the SPRUCE long-term repository (67).

## Supplementary Material

Supplementary File
