# Supplementary material for: Peatland warming strongly increases fine-root growth
Source: Proc Natl Acad Sci U S A. 2020 Jul 13;117(30):17627–34. doi: 10.1073/pnas.2003361117 (PMC7395547; doi:10.1073/pnas.2003361117)
Supplement: Supplementary File [file pnas.2003361117.sapp.pdf]

PNAS
www.pnas.org

Supplementary Information for

Peatland warming strongly increases fine-root growth

Avni Malhotra*, Deanne Brice, Joanne Childs, Jake D. Graham, Erik A. Hobbie, Holly Vander Stel, Sarah C. Feron, Paul J. Hanson and Colleen M. Iversen

*Corresponding author: Avni Malhotra

Email: avnim@stanford.edu

This PDF file includes:

Figures S1 to S8

Tables S1 to S4

SI References

www.pnas.org/cgi/doi/10.1073/pnas.2003361117

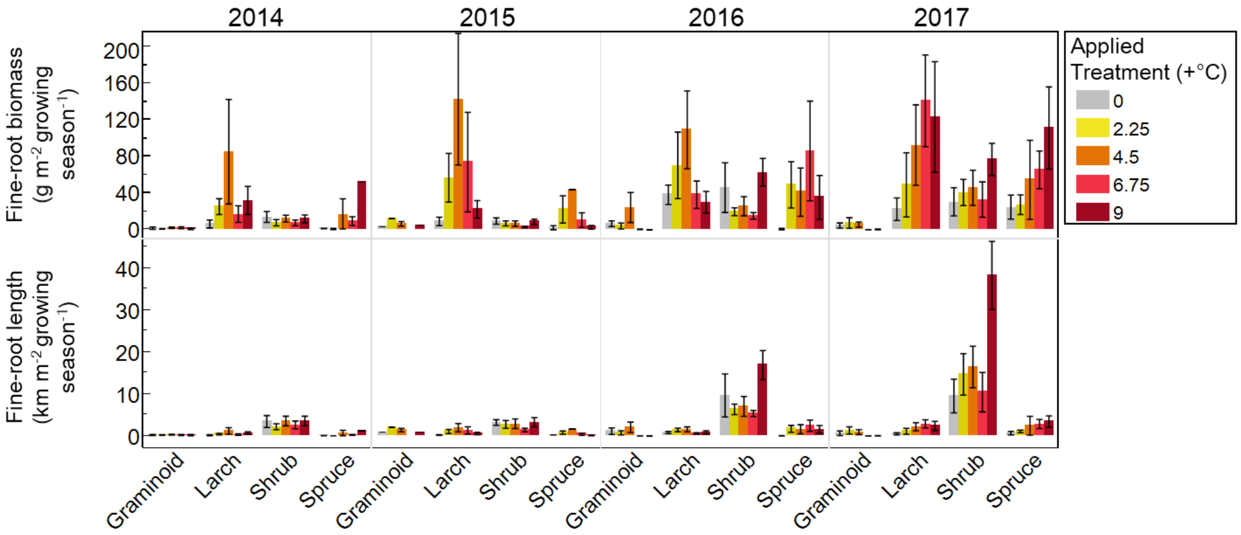

Fig. S1. Comparison of fine-root production, assessed as biomass or length, across different plant types ('larch' is *Larix laricina*, 'spruce' is *Picea mariana,* shrub and graminoid sum various species). While trees dominate over other plant types in the biomass of fine roots produced, shrubs dominate in length. Each of the growth estimates are for the June-October period for each year and applied warming treatment. Standard error bars represent variability between hummock and hollow in all the years. In 2016 and 2017, the error bars also include variability from the CO_2 treatment. Our focus on shrub length in the main manuscript provides a better measure of resource acquisition capacity (while biomass would be more of an 'investment' measure).

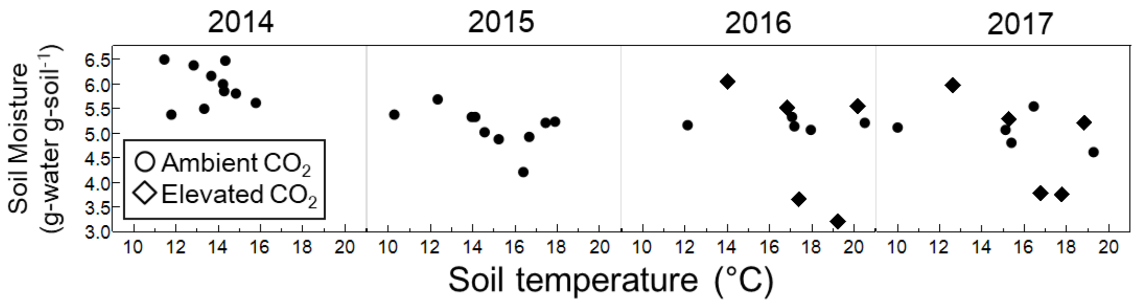

Fig. S2. No correlation between measured soil temperature (at 10 cm depth) and soil moisture (from 0 to 30 cm depths). The range of soil temperatures represents the applied temperature treatments of + 0, 2.25, 4.5, 6.75 and 9 °C.

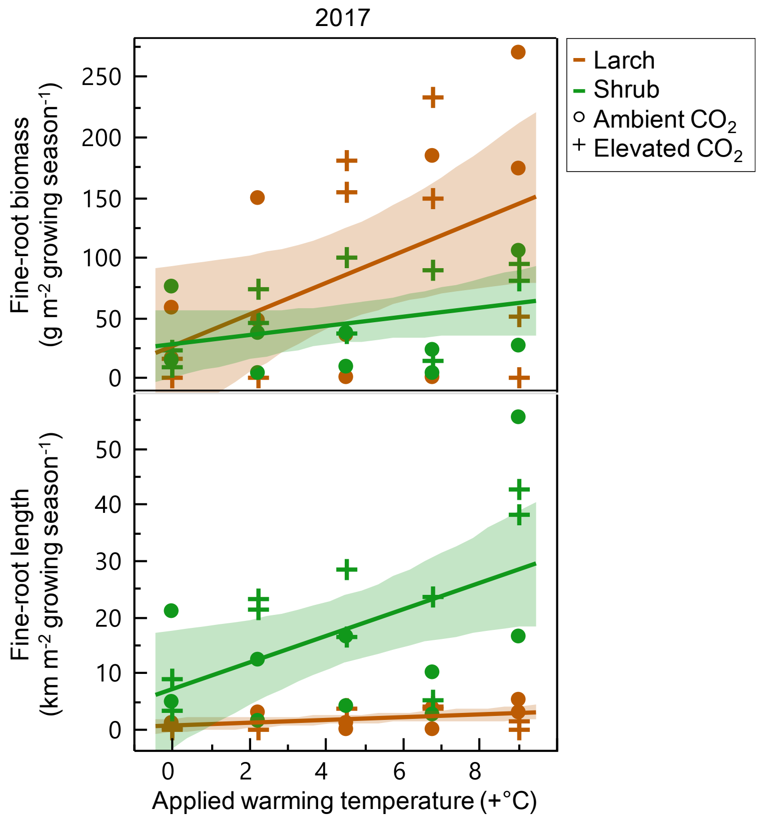

Fig. S3. Differing fine-root strategies between shrubs and larch. While shrubs significantly increased fine-root length growth, larch increased fine-root biomass growth in response to warming. The slopes of length response of shrub and larch are 2.37 and 0.25 km m^{-2} applied $°C^{-1}$ ($p<0.05$), respectively while the slopes biomass are 3.80 (non-significant) and 13.09 ($p<0.05$) g m^{-2} applied $°C^{-1}$, respectively. Similar trends are seen against measured soil temperature and moisture. Trends were also similar in the other years but 2017 had the strongest response (see Fig. S1 for other years).

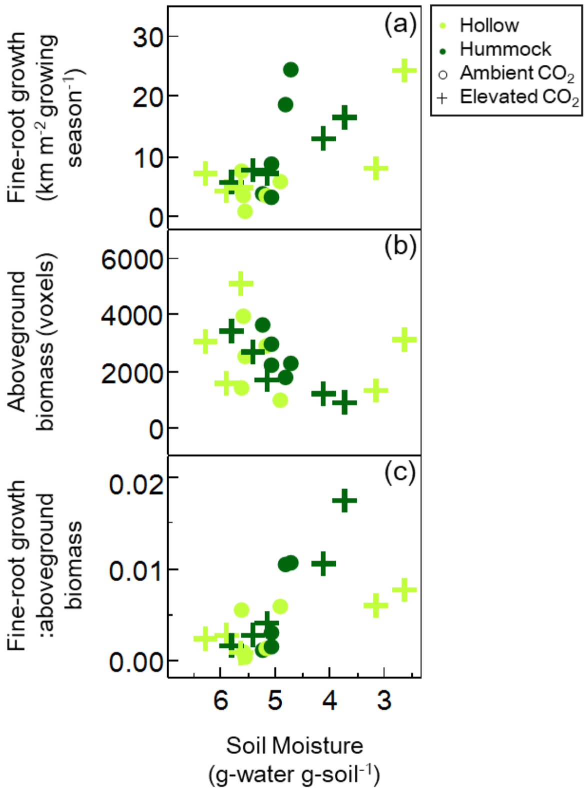

Fig. S4. The relative amount of shrub fine-root to aboveground allocation increased with drying in the 2016 growing season (inferred from ratios of shrub fine-root length production to shrub aboveground biomass). We saw the following trends with soil drying: (a) Increasing fine-root growth; (b) Decreasing aboveground biomass; and (c) Increasing fine-root:aboveground biomass with drying. Aboveground biomass was canopy volume derived from terrestrial laser scanning measurements and is expressed in voxel units (representing a total canopy volume for shrubs within a 0.5-m radius of where the ingrowth cores were located). Mixed-effects models with soil moisture and CO_2 treatment as fixed effects and topography as a random effect suggested that both fine-root growth and the ratio of fine-root growth to aboveground biomass increased significantly with soil drying (Table S3).

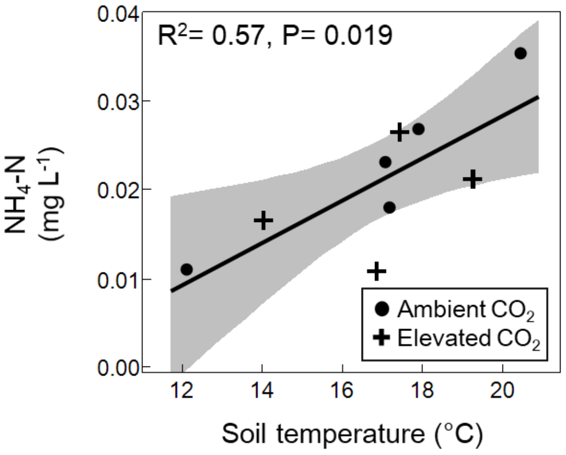

Fig. S5. Warming increases nutrient availability (inferred from ion-exchange resin data). A linear regression analysis with soil temperature and June-October 2016 average NH_4-N concentration suggested more nutrients with warming at SPRUCE (one outlier that was 0.12 mg/L NH_4-N was removed from the analysis)

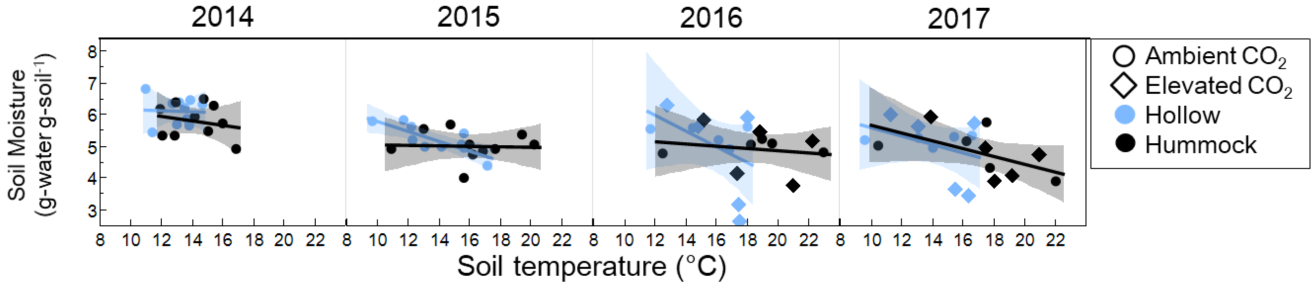

Fig. S6. Hollow soil moisture declines more than hollow with increasing soil warming. In 2015, for example, hollow slope = -0.15, R^2= 0.66, P= 0.0078, while hummock regression was not significant (slope = -0.01, R^2= 0.009, P= 0.79). This supports the notion that hollow fine roots may respond more strongly to warming than hummocks due to an increase in hollow aerobic space for roots to grow into.

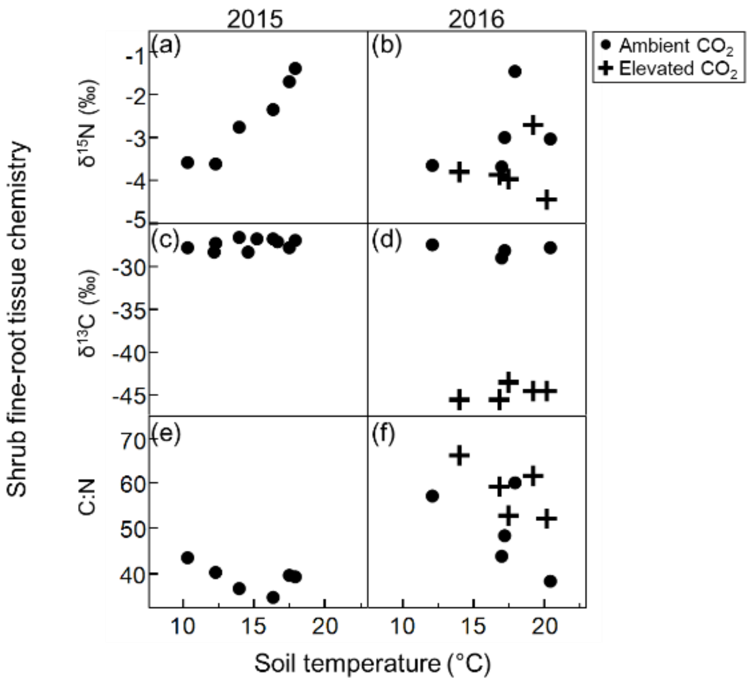

Fig. S7. Trends in shrub fine-root chemistry in 2015 and 2016 growing seasons. Sample size was lower than 10 plots (hummock and hollow were averaged) when root material was insufficient for a chemical analysis. Significant relationships with soil temperature (bivariate regressions) were found in (a) $\delta^{15}N$ from 2015 (R^2= 0.93, P= 0.0075) and (d) $\delta^{13}C$ in elevated [CO_2] plots of 2016 (R^2= 0.88, P= 0.02).

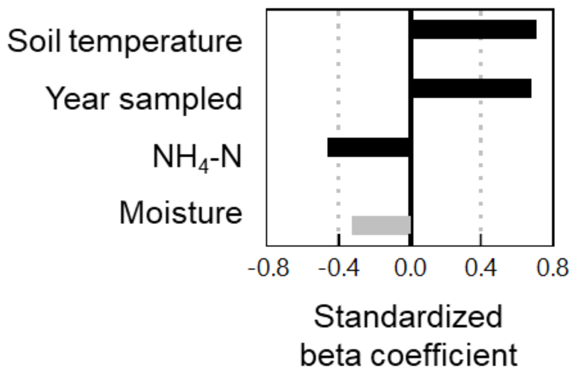

Fig. S8. Multiple regression model (R^2= 0.82, P= 0.0009) to explore predictors of shrub fine-root tissue $\delta^{15}N$ from both 2015 and 2016 June-October seasons. Black bars represent significant standard beta coefficients for each explanatory variable (relative effect of predictor on predicted variable) and the gray bars were non-significant in the model.

Table S1. Slopes of soil moisture or soil temperature increase between 2014 and 2017 when predicting log-transformed fine-root length using soil moisture or temperature. Each reported slope (p-value in parentheses; ns= non significant) is from a single bivariate linear regression run either by microtopography (n = 5; often ns due to low sample size) or plot-scale (n = 10). Since the soil moisture measurement is gravimetric water content a negative slope represents more root growth with drier soils. Table S2 shows a similar important role of year in a full multiple regression model.

	Year	By microtopography		Plot-scale
Soil moisture		Hummock	Hollow	
	2014	ns	ns	-1.07 (0.01)
	2015	ns	ns	ns
	2016	-0.55 (0.02)	-0.35 (0.03)	-0.41 (0.002)
	2017	-0.71 (0.02)	-0.67 (0.03)	-0.73 (0.001)
Soil temperature				
	2014	ns	ns	ns
	2015	ns	ns	ns
	2016	ns	ns	ns
	2017	ns	0.23 (0.03)	0.14 (0.006)

Table S2. Full mixed-effects model at the plot scale to explain variation in the fine-root length production (log-transformed). This table differs from table 1 in that it includes all possible predictors and their interactions and uses year as a fixed rather than random effect. Year is a four-level categorical variable and therefore the output shows slopes of three levels that should be interpreted relative to the fourth level (2017). Topography (hummock or hollow) is a random effect. Significant effects are marked with *.

		**Model Adj. R^2**	**Slope Estimate**	**Std Error**	**Degrees of freedom of denominator**	**P-value**	**% of random effect variation explained**
		0.65					
Fixed effects	Intercept		5.33	1.32	37.81	0.0003	
	Soil temperature		-0.04	0.05	64.6	0.4643	
	Soil moisture*		-0.54	0.15	64.02	0.0007	
	CO_2 treatment		-0.06	0.12	64.03	0.6221	
	CO_2 treatment x Soil T		0.03	0.04	64.05	0.3625	
	Year (2014)		-0.16	0.25	64.19	0.5189	
	Year (2015)*		-0.9	0.17	64.01	<0.0001	
	Year (2016)*		0.38	0.18	64.18	0.0391	
	Year (2014) x Soil T		-0.05	0.09	64.01	0.5387	
	Year (2015) x Soil T		0.03	0.06	64	0.5877	
	Year (2016) x Soil T		-0.01	0.06	64.01	0.8994	
	Year (2014) x Soil M		-0.29	0.28	64.08	0.3051	
	Year (2015) x Soil M		0.3	0.31	64	0.3395	
	Year (2016) x Soil M		0.1	0.2	64.05	0.6093	
Random effect	Topography						44.8

Table S3. Mixed-effects models of log-transformed shrub fine-root length production and aboveground biomass (as inferred from terrestrial lidar scans) to investigate the influence of drying. Soil temperature is non-significant if included in these models and was removed to specifically quantify the effect of drying. Soil moisture, and CO_2 treatment (ambient or elevated) are the fixed effects while topography is the random effect in the model (*n*= 20 in each model). Significant effects are marked with *. Corresponding data are shown in Fig. S4.

Predicted variable			Model Adj. R^2	Estimate	Std Error	Degrees of freedom of denominator	P-value	% of random effect variation explained
**Shrub fine-root growth**			0.37					
		Intercept		4.1	0.8	16.9	0.0001	
	Fixed effects	Soil moisture*		-0.4	0.2	15.0	0.0158	
		CO_2 treatment		-0.1	0.1	16.0	0.3413	
	Random effects	Topography						15.7
**Shrub fine-root growth:aboveground biomass**			0.37					
		Intercept		4.3	1.5	16.9	0.0011	
	Fixed effects	Soil moisture*		-0.6	0.2	16.0	0.0089	
		CO_2 treatment		-0.1	0.2	16.0	0.4742	
	Random effects	Topography						9.9
**Shrub aboveground biomass**			NS					

Table S4. Global Climate Models (GCMs) used in Figure 3 (Extrapolation of potential shrub fine root length production per degree warming (RCP 4.5) for North American peatlands).

Institute	Full model acronym	Resolution	Main reference
Max Planck Institute for Meteorology	MPI-ESM-LR	1.9° x 1.9°	Giorgetta et al., 2013
Canadian Centre for Climate Modelling and Analysis	CanESM2	2.8° x 2.8°	Chylek, P., et al., 2011
BOM (Bureau of Meteorology, Australia)	ACCSESS1-3	1.3° x 1.9°	Dix et al., 2013
Institute for Numerical Mathematics (INM)	INMCM4	1.5° x 2°	Volodin et al., 2010
Meteorological Research Institute, Tsukuba	MRI-CGCM3	1.1° x 1.1°	Yukimoto et al., 2012
JAMSTEC (Japan Agency for Marine-Earth Science and Technology)	MIROC-ESM-CHEM	2.8° x 1.7°	Watanabe et al.,2011
CSIRO-QCCCE-CSIRO-Mk3-6-0	CSIRO-Mk3.6.0	1.9° x 1.9°	Collier et al., 2011
Institut Pierre Simon Laplace	IPSL-CM5A-MR	1.3° × 2.5°	Dufresne et al., 2013
Met Office Hadley Centre ESM	HadGEM2-AO	1.25° x 1.8°	Baek et al, 2013
Centro Euro-Mediterraneo sui Cambiamenti Climatici Climate Model	CMCC	0.75° x0.75°	Scoccimarro et al, 2011

SI References

Baek, H. J., Lee, J., Lee, H. S., Hyun, Y. K., Cho, C., Kwon, W. T., ... & Lee, J. (2013). Climate change in the 21st century simulated by HadGEM2-AO under representative concentration pathways. Asia-Pacific Journal of Atmospheric Sciences, 49(5), 603-618.

Chylek, P., J. Li, M. K. Dubey, M. Wang, and G. Lesins. "Observed and model simulated 20th century Arctic temperature variability: Canadian earth system model CanESM2." Atmospheric Chemistry and Physics Discussions 11, no. 8 22893-22907 (2011).

Collier, M. A., Jeffrey, S. J., Rotstayn, L. D., Wong, K. K., Dravitzki, S. M., Moseneder, C., ... & El Zein, A. (2011, December). The CSIRO-Mk3. 6.0 Atmosphere-Ocean GCM: participation in CMIP5 and data publication. In International Congress on Modelling and Simulation–MODSIM.

Dix, M., Vohralik, P., Bi, D., Rashid, H., Marsland, S., O'Farrell, S., Uotila, P., Hirst, T., Kowalczyk, E., Sullivan, A. and Yan, H. The ACCESS coupled model: documentation of core CMIP5 simulations and initial results. Aust. Meteorol. Oceanogr. J, 63(1) pp.83-99 (2013).

Dufresne, J. -L. et al. Climate change projections using the IPSL-CM5 Earth System Model: from CMIP3 to CMIP5. Clim. Dyn. 40, 2123-2165 (2013).

Giorgetta, M. A. et al. Climate and carbon cycle changes from 1850 to 2100 in MPI-ESM simulations for the Coupled Model Intercomparison Project phase 5. J. Adv. Model. Earth Syst. 5, 572-597 (2013).

Scoccimarro E., S. Gualdi, A. Bellucci, A. Sanna, P.G. Fogli, E. Manzini, M. Vichi, P. Oddo, and A. Navarra, 2011: Effects of Tropical Cyclones on Ocean Heat Transport in a High Resolution Coupled General Circulation Model. J. Clim. 24, 4368-4384.

Volodin, E. M., Dianskii, N. A., & Gusev, A. V. Simulating present-day climate with the INMCM4.0 coupled model of the atmospheric and oceanic general circulations. Izvestiya, Atmospheric and Oceanic Physics, 46(4): 414-431 (2010).

Watanabe, M. et al. Improved climate simulation by MIROC5: Mean states, variability, and climate sensitivity. J. Clim. 23, 6312-6335 (2010).

Yukimoto, S., Adachi, Y., Hosaka, M., Sakami, T., Yoshimura, H., Hirabara, M., Tanaka, T.Y., Shindo, E., Tsujino, H., Deushi, M. and Mizuta, R. A new global climate model of the Meteorological Research Institute: MRI-CGCM3—model description and basic performance—. Journal of the Meteorological Society of Japan. Ser. II, 90, pp.23-64 (2012).
